# Three surgical techniques using different energy sources for anatomical endoscopic enucleation of the prostate

**DOI:** 10.3389/fsurg.2025.1668140

**Published:** 2025-09-12

**Authors:** Xin Yi, Gongliang Zhu, Biyu Zhu, Junrong Zou, Jingyi Zhu, Xiaofeng Zou, Guoxi Zhang

**Affiliations:** ^1^ First Clinical College, Gannan Medical University, Ganzhou, China; ^2^ Department of Urology, First Affiliated Hospital of Gannan Medical University, Ganzhou, China; ^3^ Institute of Urology, Gannan Medical University, Ganzhou, China; ^4^ Jiangxi Engineering Technology Research Center of Calculi Prevention, Ganzhou, China

**Keywords:** benign prostatic hyperplasia, enucleation of the prostate, bipolar enucleation of prostate, holmium laser enucleation of prostate, thulium laserenucleation of prostate

## Abstract

Although transurethral resection of the prostate (TURP) remains the gold standard for the surgical treatment of benign prostatic hyperplasia (BPH), this approach is associated with a high recurrence rate and numerous postoperative complications. Recently, advancements in equipment and surgical techniques have led to the increased clinical application of anatomical endoscopic enucleation of the prostate (AEEP). The primary devices used in AEEP include bipolar plasma systems, holmium lasers, and thulium lasers. This article presents a narrative review evaluating the performance of these three energy sources in surgery and provides a comparative analysis of their advantages and disadvantages.

## 1 Introduction

BPH is the most prevalent benign neoplasm responsible for lower urinary tract symptoms (LUTS) in middle-aged and elderly men. Its pathogenesis is attributed to uncontrolled proliferation of stromal and epithelial cells in the transition zone (TZ) and periurethral region, ultimately leading to benign prostatic enlargement (1). The size of the prostate in an adult male is about 25 g (2), and its size increases to varying degrees with age. BPH can cause varying degrees of bladder outlet obstruction (BOO), primarily due to urethral compression induced by prostate enlargement, leading to impaired urinary flow. Clinical manifestations may include lower urinary tract symptoms (LUTS), infections, urinary retention, and other complications (3). Although LUTS can result from a wide range of etiologies, voiding LUTS are most commonly attributed to BOO secondary to BPH (4). To manage BOO caused by BPH, both pharmacological and surgical interventions are well-established clinical approaches. For patients with mild BPH symptoms, drug therapy is considered the first-line treatment option. Available pharmacological regimens include: α-adrenergic receptor blockers, 5α-reductase inhibitors, phosphodiesterase-5 (PDE5) inhibitors, β3-agonists, anticholinergic agents, and combination therapies involving these different drug classes (5). However, while medications can alleviate a variety of urinary symptoms caused by BPH, their effectiveness is limited in cases of significantly enlarged prostates. Additionally, the side effects of pharmacological treatments and the financial burden associated with long-term medication use present considerable challenges for many BPH patients. Furthermore, disease progression often continues gradually in many patients despite receiving drug therapy. In this context, surgical intervention has emerged as an effective therapeutic option for significantly improving urinary obstruction in BPH patients (6). Currently, surgical treatment for BPH is increasingly shifting toward minimally invasive approaches. Clinicians are transitioning from traditional open prostatectomy to endoscopic procedures, which involve the use of specialized instruments inserted transurethrally to remove excess prostate tissue. This shift not only enhances surgical precision but also significantly reduces postoperative discomfort for patients. At present, although TURP is still regarded as the widely recognized gold standard for the surgical treatment of BPH (7), it is still associated with a range of postoperative complications and surgical contraindications that can negatively impact patients' quality of life after surgery. With the ongoing advancement of medical equipment and surgical techniques, plasma devices and lasers have been increasingly utilized in the surgical treatment of BPH, demonstrating their unique advantages in achieving better operative outcomes. Additionally, the preferred surgical approach has gradually transitioned from TURP to TUEP (8). Therefore, we herein discuss the differences between bipolar plasma technology and laser technology in the surgical treatment options for BPH. This discussion will clarify the selection criteria for these surgical approaches in various scenarios and present the current clinical application status of these techniques.

## 2 Technology overview and working principle

### 2.1 Bipolar plasma kinetic

The cutting system of bipolar plasma devices consists of two electrodes: a radiofrequency electrode and a return electrode, both integrated within the resectoscope loop. This configuration allows the current to flow directly through the loop, thereby significantly reducing the current pathway through the patient's body via a neutral plate and consequently minimizing potential interference with implanted cardiac pacemakers (9). In contrast to monopolar devices, which require non-ionic irrigation solutions as the conductive medium in the bladder—where these hypotonic solutions carry a significant risk of systemic absorption, potentially leading to water intoxication or even fatal outcomes—bipolar plasma technology operates effectively using isotonic normal saline. This approach substantially reduces the incidence of transurethral resection syndrome (TURS) while simultaneously allowing for extended procedure durations to manage larger prostate volumes (10). In the surgical management of BPH, bipolar plasma devices equipped with plasma resectoscope loops offer an expanded cutting surface area and operative range. This system demonstrates a relatively simplified operation compared to alternative devices, leading to higher adoption rates in many primary care hospitals. Depending on the surgical indications, this technology can be utilized for either transurethral resection of the prostate (TURP) or bipolar transurethral enucleation of the prostate (B-TUEP) (11).

### 2.2 Holmium laser enucleation of the prostate (HoLEP)

The Yttrium Aluminum Garnet (YAG) crystal serves as a widely utilized solid-state laser medium in holmium laser systems. The holmium laser emits pulsed radiation at a wavelength of 2,140 nm, which demonstrates strong water absorption characteristics. This property facilitates its clinical applications, not only in lithotripsy procedures but also in soft tissue ablation, including the surgical treatment of benign prostatic hyperplasia. The laser exhibits a shallow tissue penetration depth of 0.4 mm in prostatic tissue, resulting in both vaporization effects through photothermal ablation and coagulation of blood vessels up to 2 mm deep. These combined effects enhance intraoperative hemostasis during surgical procedures (12). The advantage of the holmium laser in benign prostatic hyperplasia surgery lies in its superior ability to manage large-volume prostate hyperplasia. It achieves adenoma resection volumes comparable to those of open prostatectomy while providing the benefits of minimally invasive transurethral surgery and promoting a more favorable postoperative recovery (13). Moreover, in the surgical management of patients requiring anticoagulant therapy, holmium laser enucleation can be performed without the need to discontinue anticoagulant medications. Although this approach may lead to prolonged postoperative hospitalization and an extended duration of indwelling catheter use, it significantly reduces the risk of thromboembolic events associated with the withdrawal of anticoagulants. This evidence underscores the enhanced safety profile of holmium laser enucleation for patients undergoing anticoagulation therapy (14). To date, an increasing number of clinicians have adopted holmium laser enucleation combined with a morcellator for prostatectomy in order to achieve improved surgical outcomes (15).

### 2.3 Thulium laser enucleation of the prostate (ThuLEP)

The thulium laser emits continuous-wave visible light at a wavelength of 2,013 nm, which is characterized by high water absorption and a penetration depth of 0.25 mm. Currently, the predominant thulium laser systems include Tm:YAG (Revolix) and Tm-fiber (Vela XL). Due to its relatively lower power and higher cutting efficiency, the thulium laser has been widely adopted in urological procedures, such as stone treatment and prostate surgery (16). The active medium in thulium laser devices comprises thulium-doped silica fiber matrices. This design enables efficient air cooling, effectively mitigating thermal buildup during extended operation and reducing mechanical disturbances typically associated with conventional cooling systems (17). The thulium laser system employs a diode-based pumping mechanism, which is different from the flashlamp-pumped holmium laser configuration. This design requires less power input while maintaining a stable high-power output, thereby eliminating the need for additional fiber cooling measures during operation (18). The thulium laser's continuous-wave emission mode results in shallower tissue penetration (0.2–0.3 mm), allowing for immediate tissue vaporization. With its emission wavelength closely matching the water absorption peak (approximately 2,013 nm), this laser system provides precise cutting control in soft tissue surgeries, such as prostate procedures, which may contribute to reduced collateral tissue damage (19). The thulium laser system, equipped with quartz fiber, demonstrates lower acoustic emissions during operation and enhanced portability compared to conventional systems. In surgeries for benign prostatic hyperplasia, its tissue interaction mechanism relies primarily on direct laser cutting rather than vapor bubble separation. Combined with its characteristically shallow penetration depth of 0.2–0.3 mm, this system exhibits a superior capability for preserving peri-prostatic soft tissues. Clinical evidence suggests that this technological profile may correlate with improved functional outcomes, including the preservation of erectile function, when compared to alternative surgical modalities (20).

## 3 Advantages and disadvantages of the three surgical instruments

### 3.1 B-TUEP

#### 3.1.1 Advantages

Since the initial description of transurethral prostate enucleation in 1989, prostate enucleation techniques have gained popularity as a surgical option for managing BPH (21). In 2006, Neill et al. utilized a bipolar energy system as an alternative to laser energy, defining the procedure as bipolar enucleation of the prostate (BipolEP) (22). The bipolar system generates localized, high-density current exclusively at the resection loop, preventing current from passing through the patient's body. This significantly reduces the complications associated with monopolar systems. Consequently, it has been widely adopted in clinical practice across major hospitals in Asia (23). Meanwhile, some scholars suggest that bipolar plasma surgery may provide lower operational costs compared to other surgical instruments, potentially alleviating the financial burden on patients (24). Additionally, B-TUEP achieves a more comprehensive adenoma resection compared to B-TURP, demonstrating advantages in alleviating bladder outlet obstruction and enhancing prostate tissue removal rates. These factors may contribute to lower recurrence rates of BPH (25). Furthermore, regarding the learning curve, B-TUEP demonstrates a shorter training period compared to other laser surgeries and is comparable to conventional TURP. This feature may allow junior surgeons to attain proficiency more quickly and perform the procedure independently, potentially providing clinicians with a safer and more efficient surgical option (26). Regarding surgical efficacy, studies with follow-up data ranging from 6 to 12 months indicate that B-TUEP demonstrates significantly higher Qmax and lower IPSS scores compared to monopolar TURP during this postoperative period. Additionally, B-TUEP shows superior performance in reducing the incidence of TURS relative to monopolar TURP, while no significant differences were observed in complications such as retrograde ejaculation and urinary tract infections (27). For large-volume prostate surgery, B-TUEP demonstrates comparable efficacy to open transvesical prostatectomy in both short-term and mid-term outcomes, while requiring shorter irrigation durations and hospital stays. These findings indicate that B-TUEP achieves similar resection outcomes to open surgery while providing the advantages of a minimally invasive approach, leading to improved postoperative recovery (28). Moreover, bipolar plasma devices demonstrate lower equipment costs compared to alternative systems, which may partially account for their wider clinical adoption in comparison to laser-based technologies (29).

#### 3.1.2 Disadvantages

In comparison to laser enucleation for large-volume prostate surgery, bipolar plasma systems do not demonstrate a significant advantage in operative efficiency. Research evidence indicates that laser procedures exhibit superior performance in both operative duration and hemoglobin loss, highlighting certain limitations of bipolar plasma technology in large prostate enucleation procedures (30). Additionally, B-TUEP is associated with longer postoperative hospital stays compared to laser prostate enucleation procedures. This difference may be attributed to the superior hemostatic efficiency of laser systems, which can lead to shorter durations of catheterization and reduced bladder irrigation requirements following laser surgery (31). Several studies comparing postoperative recovery patterns among different BPH surgical treatments have reported that bipolar procedures require longer convalescence periods than laser techniques. This extended recovery duration, in turn, prolongs hospitalization, which may negatively impact patients' quality of life (32). The larger physical dimensions of bipolar plasma electrodes, in comparison to laser fibers, inevitably compromise the surgeon's field of view, which may subsequently affect surgical efficiency. Additionally, when addressing BPH cases complicated by concurrent bladder stones, bipolar plasma systems lack integrated lithotripsy capabilities, potentially requiring additional instruments for stone fragmentation, which may accelerate device wear. In contrast, laser enucleation systems allow for simultaneous stone management, thereby enhancing procedural efficiency and reducing operative time (33).

### 3.2 HoLEP

#### 3.2.1 Advantages

HoLEP has been established as a size-independent surgical treatment for benign prostatic hyperplasia. Clinical studies demonstrate that it has comparable therapeutic efficacy to open prostatectomy (OP), while offering advantages such as shorter catheterization duration, reduced intraoperative blood loss, and faster postoperative recovery, all attributable to its minimally invasive nature (13, 34). The holmium laser generates distinctive plasma bubbles at the fiber tip when used in a saline medium. These bubbles are recognized as rapidly expanding vapor bubbles that exhibit dilatational effects. Upon expansion and subsequent collapse, these bubbles produce localized cavitational energy, generating pressure waves that can induce rapid tissue collapse and ablation. This mechanism ultimately facilitates blunt tissue dissection. As a result, operators can maintain the fiber at a distance of 3–4 mm from the cutting plane, allowing for the creation of a well-defined anatomical plane between the adenoma and the surgical prostatic capsule (29, 33). Comparative studies have shown that HoLEP is associated with better outcomes compared to B-TUEP, including less hemoglobin loss and a lower incidence of surgical complications. These findings indicate potential advantages of HoLEP in terms of both therapeutic efficacy and procedural safety (35). Several studies have reported that HoLEP yields superior outcomes compared to TURP in terms of surgical complications, particularly intraoperative bleeding. This advantage may be attributed to two key mechanisms: First, the combined vaporization and coagulation effects of laser energy during enucleation facilitate simultaneous anatomical dissection and hemostasis of the prostatic vasculature. Second, unlike the repetitive cutting motion of TURP, which creates multiple fresh vascular openings, HoLEP employs a single-plane coagulation technique at the level of the prostatic capsule, resulting in more effective hemostasis while preserving optimal surgical visibility (36). These findings demonstrate the superior hemostatic performance of the holmium laser compared to bipolar plasma and other surgical instruments, which may explain the particularly favorable outcomes of HoLEP in patients requiring preoperative anticoagulation therapy (37). A meta-analysis revealed that although HoLEP requires a longer operative time, it demonstrates superior postoperative outcomes, including a shorter duration of catheter indwelling and reduced hospital stays compared to alternative procedures. Patients undergoing Holmium laser surgery experienced significantly shorter recovery periods (36). A 10-year follow-up study demonstrated that HoLEP provides lasting improvement in BOO symptoms. Long-term data indicate lower recurrence rates of obstructive symptoms compared to other surgical modalities. These findings suggest favorable long-term outcomes for HoLEP in preventing the recurrence of BOO (38).

#### 3.2.2 Disadvantages

Although HoLEP offers numerous advantages in clinical applications, it also presents some significant drawbacks. Firstly, the operation of laser surgery is relatively complex, necessitating that surgeons undergo extensive skill training and accumulate experience to control postoperative complications at an ideal level (39, 40). This requirement compels surgeons to view a substantial number of surgical videos and engage in considerable surgical practice to master this skill proficiently, which, to some extent, limits the widespread adoption and promotion of laser enucleation of the prostate. Secondly, holmium laser equipment necessitates the use of specialized laser resection sheaths, optical fibers, and morcellation devices, which inevitably increases the economic cost of the procedure. Additionally, the relatively longer duration of laser surgery further contributes to the overall economic expense (41). Moreover, because laser surgery requires the complete enucleation of the prostate along the surgical capsule, an additional morcellation device is necessary to fragment and remove the enucleated tissue. This requirement increases the number of procedural steps for the surgeon. Furthermore, the safety and efficacy of the morcellation process in managing prostate tissue are critically important, as they significantly contribute to reducing surgical complications, such as bleeding and bladder perforation (42).

### 3.3 ThuLEP

#### 3.3.1 Advantages

The thulium laser, a novel energy source recently adopted by many researchers for BPH surgery, exhibits a lower tissue penetration depth compared to other lasers (43). With a penetration depth of only 0.1–0.2 mm in prostatic tissue, the thulium laser allows for more precise cutting and controlled ablation (44). Furthermore, studies have demonstrated that laser enucleation of the prostate offers higher enucleation efficiency, indicating that surgeons may adapt to the technique more quickly, resulting in a shorter learning curve (45). Due to its precise cutting capability, minimal tissue penetration depth, and advanced hemostatic properties, the thulium laser not only improves surgical visibility but also protects surrounding tissues, thereby reducing intraoperative damage (44). Furthermore, the thulium laser, with its unique continuous-wave output characteristics, minimizes damage to critical peri-prostatic tissues, thereby better preserving their normal anatomical structure. In contrast, the pulsed output of the holmium laser results in tissue fragmentation and exerts greater mechanical stress on the tissues intended for preservation. In this context, the thulium laser offers significant advantages in functional tissue preservation, such as maintaining patients' ejaculatory function (46). A recent study found that among laser devices, the thulium laser has a slightly shorter learning curve compared to the holmium laser. This allows surgeons to become proficient in its use for relevant surgical procedures in a shorter amount of time (47). Moreover, a meta-analysis comparing holmium and thulium lasers in prostate enucleation demonstrated that the thulium laser was associated with less hemoglobin reduction than the holmium laser. This suggests superior hemostatic performance with the thulium laser, resulting in reduced intraoperative blood loss and consequently lesser hemoglobin decline—potentially attributable to its unique vaporization-based mechanism, which enhances vascular coagulation (48). Additionally, among the currently prevalent laser devices, the thulium laser generates less noise than the holmium laser, contributing to a quieter operating room environment and allowing surgical staff to work with greater focus (49).

#### 3.3.2 Disadvantages

Although the thulium laser, as a relatively new laser device, offers multiple advantages in the treatment of BPH, it still faces limitations that hinder its widespread adoption. For instance, while both holmium and thulium laser fibers are reusable, holmium laser systems are more frequently utilized in procedures such as lithotripsy and urethrotomy. This broader range of applications allows the cost of holmium laser equipment to be distributed more effectively compared to the thulium laser (48). Although thulium lasers have gradually been applied in lithotripsy surgeries, most physicians still choose holmium lasers as their first option. Moreover, the maintenance costs and equipment expenses of thulium lasers remain higher compared to other instruments. Secondly, among laser devices, the blasting effect of holmium lasers enables clearer separation of the prostatic surgical capsule than thulium lasers. However, due to the continuous wave mode of thulium lasers during tissue surface cutting, a eschar-like vision is formed on the cutting plane, which results in a longer time required to identify the surgical capsule plane during prostate enucleation surgery compared to holmium lasers. In some instances, this can increase the risk of capsular perforation due to impaired visualization of anatomical boundaries (29). Most importantly, because the popularity of thulium lasers is not as widespread as that of holmium lasers and plasma systems, there is currently no standardized large-scale data available to demonstrate the reliability and effectiveness of thulium lasers (50) (Table 1).

**Table 1:** Comparison of advantages and disadvantages of the three surgical procedures.

Surgical instruments	Advantages	Disadvantages
Bipolar plasma kinetic	It features low equipment cost, the possibility of switching to TURP at any time during the operation, and a short learning curve.	It has limitations in operations involving large-volume glands, is associated with a long postoperative recovery period, and cannot address bladder stones simultaneously.
HoLEP	It allows for clearer identification of anatomical planes during surgery, can address bladder stones simultaneously, and is associated with a relatively shorter postoperative hospital stay.	It has a relatively long learning cycle and requires the additional use of a dedicated fragmentation device to remove the gland.
ThuLEP	It enables precise intraoperative manipulation to better protect surrounding tissues and produces relatively lower noise during surgery.	It is associated with high equipment costs and difficulty in identifying anatomical planes during surgery.

## 4 Discussion

Through the continuous practice of numerous surgeons as well as innovations in equipment and technology, prostate enucleation technology has become an increasingly preferred treatment option for moderate to large-volume benign prostatic hyperplasia, and has been recognized by the European Association of Urology (EAU) and the American Urological Association (AUA) (51, 52). Meanwhile, it is acknowledged that advancements in equipment and technology have not only provided surgeons with a variety of surgical methods but have also empowered patients and their families with the right to make informed choices. Patients and their families can thoroughly evaluate surgical outcomes, complication rates, and costs to select the most appropriate surgical methods and instruments. Currently, numerous scholars, guided by established protocols, have identified the surgical indications for BPH as follows: (1) long-term bothersome lower urinary tract symptoms (LUTS), including acute urinary retention; (2) patients' desire to avoid daily medication or other treatments while achieving significant improvement in LUTS; (3) intolerance to medications, or acute/chronic renal insufficiency resulting from BPH, refractory urinary retention, recurrent urinary tract infections (UTI), long-term recurrent bladder stones, and persistent gross hematuria (53).

The clinical manifestations of BPH typically present as LUTS. Therefore, it is crucial to determine whether these symptoms in patients are indeed caused by BPH. Herein, we introduce the methods used to diagnose BPH: (1) The patient's condition can be initially assessed by reviewing their medical history, and a preliminary evaluation of patients with dysuria can be performed using a frequency-volume chart. (2) After considering that the patient's LUTS may be caused by BPH based on medical history, a comprehensive physical examination is required. Additionally, a digital rectal examination should be performed to preliminarily assess the size of the prostate and detect any irregular malignant hyperplasia. (3) Laboratory tests include prostate-specific antigen (PSA) measurement and urinalysis, as well as renal function tests for patients with long-term BPH to assess whether urinary obstruction symptoms have caused kidney damage. (4) Imaging examinations include color Doppler ultrasound of the urinary system and specialized prostate magnetic resonance imaging, which help clinicians more accurately assess the size of the patient's prostate and detect any malignant transformation (54). It is essential to differentiate BPH from other conditions with similar symptoms, such as urodynamic detrusor underactivity (DUA). Patients with DUA may also experience incomplete bladder emptying, resulting in symptoms that mimic BOO seen in BPH. Therefore, urodynamic testing is necessary to distinguish between these conditions before surgery. However, due to its invasive nature and relatively high cost, urodynamic testing has limitations in preoperative diagnosis. Consequently, it is often impossible to definitively confirm preoperatively whether male patients have BOO caused specifically by BPH. As a result, some patients with concurrent DUA or those with DUA misdiagnosed as BPH may continue to experience incomplete bladder emptying after BPH surgery (55). On this basis, the AUA guidelines also recommend that urodynamic testing be considered as an examination method for patients with BPH prior to surgery (56).

Similarly, diode lasers and green lasers, which have also been used clinically for prostate enucleation, each possess unique characteristics. Diode laser enucleation of the prostate (DiLEP) is performed using diode lasers, which are less expensive compared to other laser devices (57). The surgical approach of DiLEP is fundamentally similar to that of other laser devices. However, because the emitted light has a frequency range of 375–1,800 nm, the effects produced by different diodes vary slightly (58). Initially, semiconductor lasers were commonly used to vaporize prostatic adenomas. With advancements in equipment, it was discovered that diode lasers are also absorbed by water and hemoglobin, leading to their application in prostate enucleation. Although studies have confirmed the clinical effectiveness of DiLEP, demonstrating better surgical outcomes and a lower incidence of complications compared to TURP, there is a lack of extensive comparative data with other laser devices. Therefore, whether DiLEP offers superior results compared to the currently widely used holmium and thulium lasers remains to be determined (59). The green laser emits light at a wavelength of 532 nm, produced by combining an Nd:YAG laser with a potassium titanyl phosphate (KTP) crystal. It has a coagulation depth of only 1–2 nm and is selectively absorbed by hemoglobin. Due to the use of a 70° side-firing optical fiber—unlike those used in holmium and thulium lasers—its tissue vaporization effect is less effective compared to these two commonly used laser devices in clinical practice. As a result, it is difficult to achieve a clear visual field to distinguish anatomical planes during surgery (23). As an early laser device, studies have found that green laser enucleation of the prostate (GreenLEP) requires less time than HoLEP during prostate enucleation surgeries (60). Previous reports have shown that green lasers provide favorable therapeutic effects and lead to fewer postoperative complications during surgery, especially in patients taking anticoagulants. With ongoing advancements, the maximum power of green lasers has increased from the initial 80–180 W, reflecting improvements in laser efficiency and surgical outcomes. However, there are still relatively few surgical studies on the latest high-power green laser devices, indicating that the feasibility of these new high-power lasers remains to be fully validated (61).

Although the guidelines of the European Association of Urology and the American Urological Association still consider open simple prostatectomy (OSP) the gold standard for large-volume prostates (>80 ml), laparoscopic simple prostatectomy (LSP) and robot-assisted simple prostatectomy (RASP) are increasingly less favored as invasive treatment options for BPH. This trend reflects clinicians' growing preference for minimally invasive surgery. Both LSP and RASP are associated with a higher risk of surgical and perioperative complications compared to transurethral procedures, as well as longer hospital stays (62). Although the EAU guidelines have gradually recognized the use of HoLEP and B-TUEP for surgeries involving large-volume prostates—since they achieve nearly equivalent surgical outcomes with a lower incidence of postoperative complications—LSP has an advantage over HoLEP in terms of the learning curve, making it easier for beginners to master. However, regarding cost, both LSP and RASP are associated with higher expenses compared to B-TUEP and other transurethral prostate surgeries, leading patients to prefer surgical options that are more minimally invasive and less costly (63).

In patients with a history of prostate surgery, residual glandular tissue resulting from incomplete resection may lead to BPH recurrence and the subsequent re-emergence of LUTS. Although many physicians prefer TURP in such cases, recent studies have indicated that AEEP is both safe and effective for these patients. For those with recurrent BPH, the postoperative efficacy of HoLEP demonstrates an improvement nearly comparable to that of the initial surgery. Due to altered prostate anatomy during reoperation for recurrent BPH, tissue planes are less distinct than in the initial procedure. Consequently, B-TUEP becomes more challenging in these patients because of less precise cutting and limited manipulation accuracy. Given the limited sample size of data on ThuLEP for secondary surgery in recurrent BPH, there is insufficient evidence to confirm the surgical efficacy of the thulium laser in this context, warranting further studies (64). Nevertheless, regardless of the surgical approach, reoperation for recurrent BPH is more difficult and demanding for surgeons compared to the initial surgery, necessitating that clinicians possess substantial surgical experience to perform the secondary procedure successfully.

**Figure 1:**
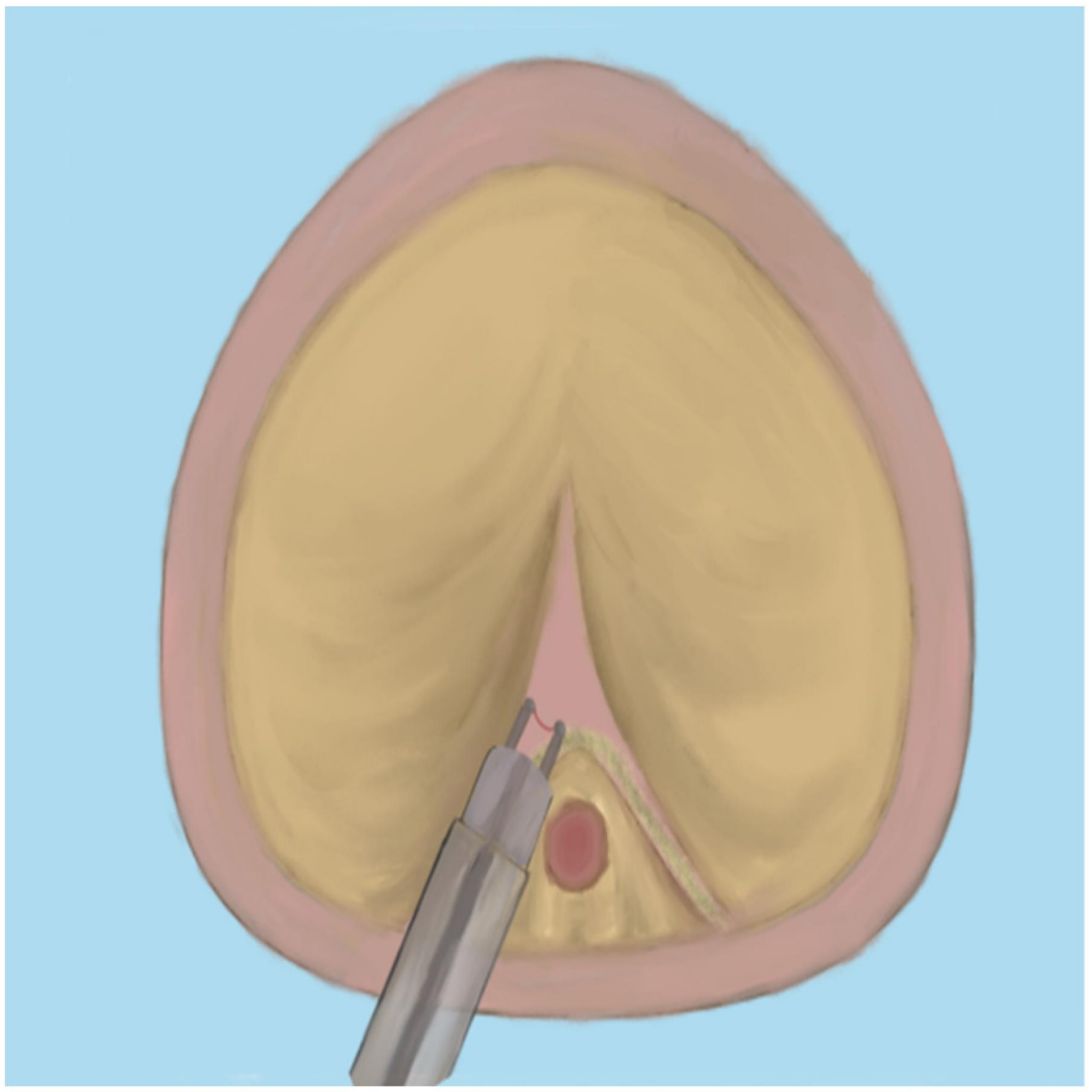
Verumontanum processing.

**Figure 2:**
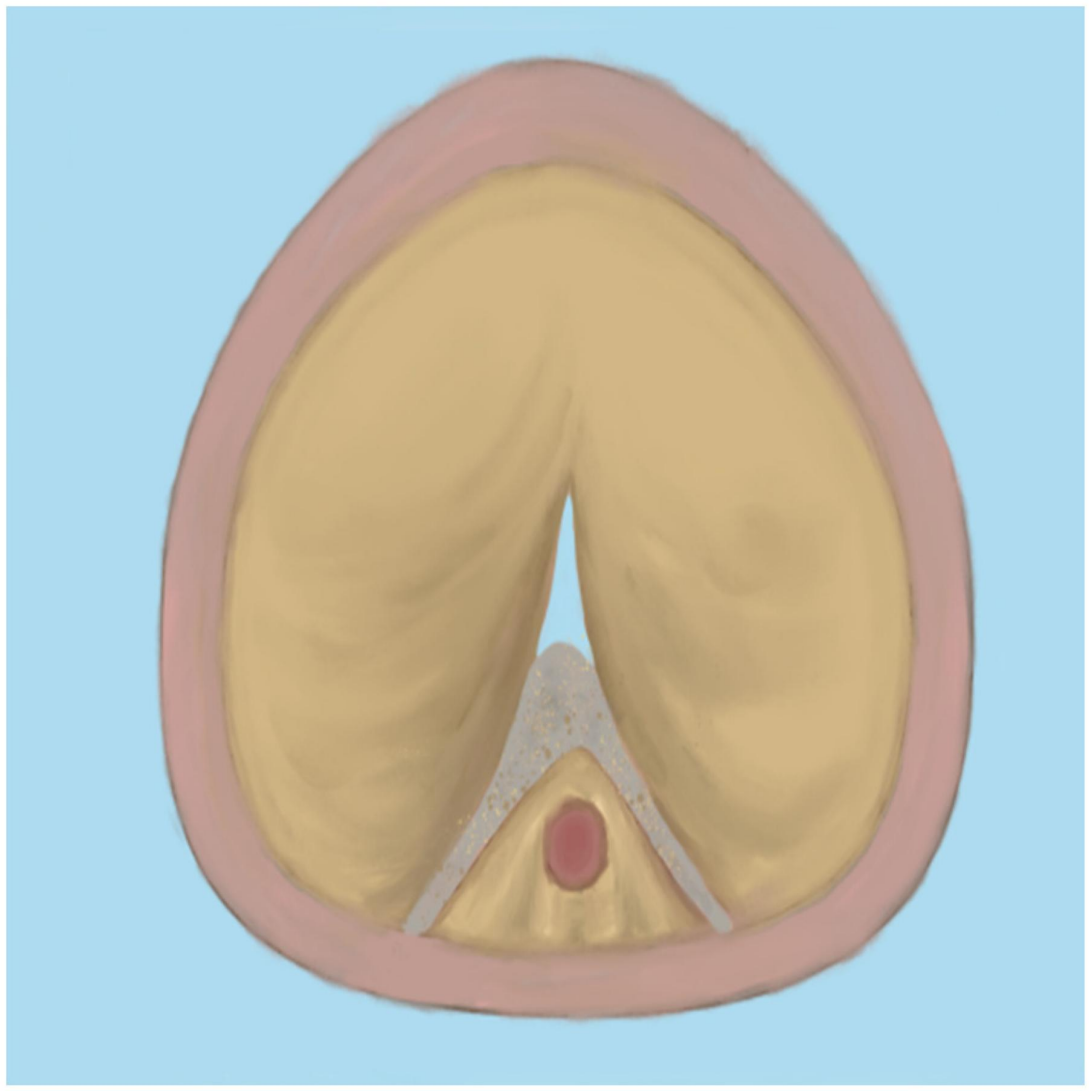
After enucleation of the median lobe.

**Figure 3:**
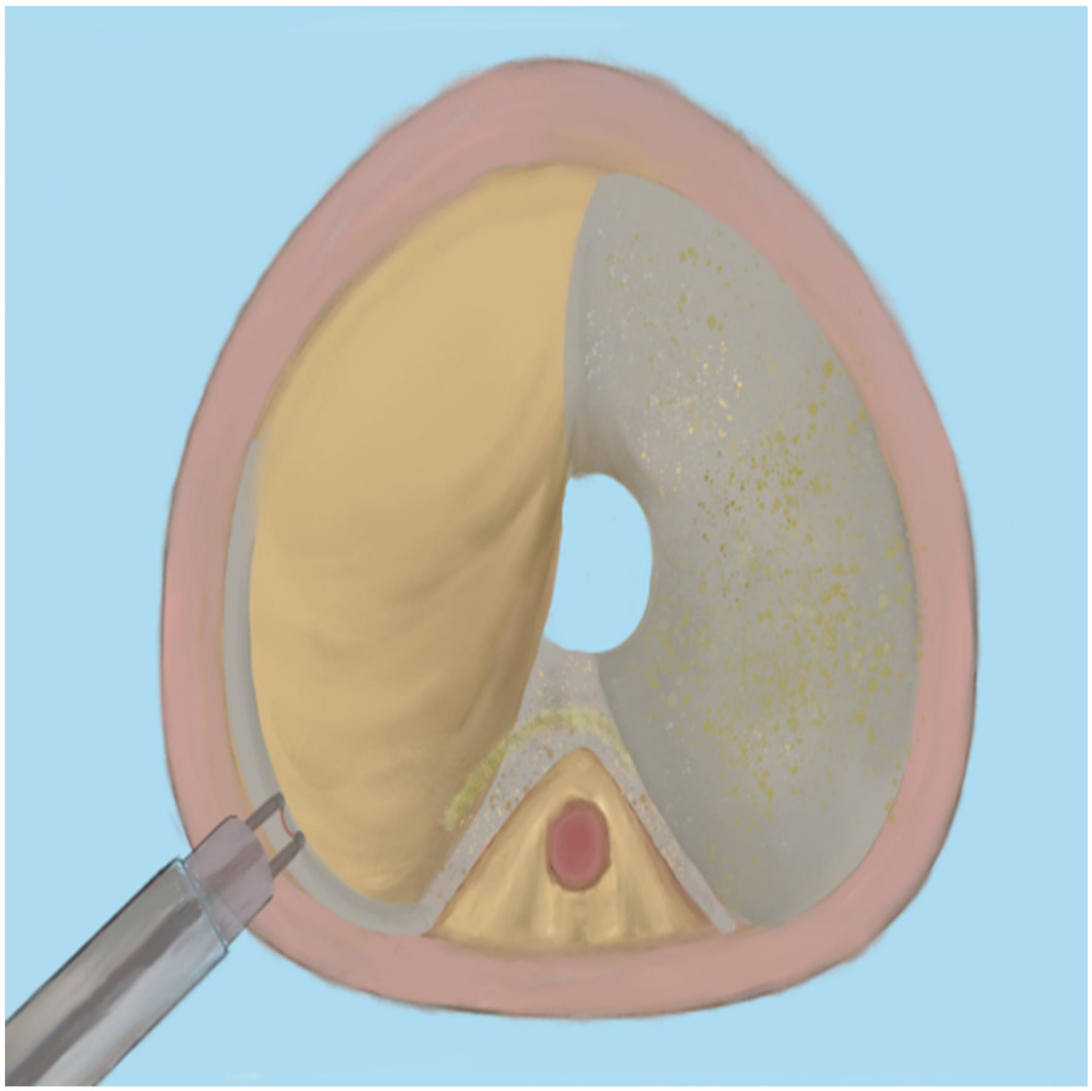
Enucleate the right lobe.

**Figure 4:**
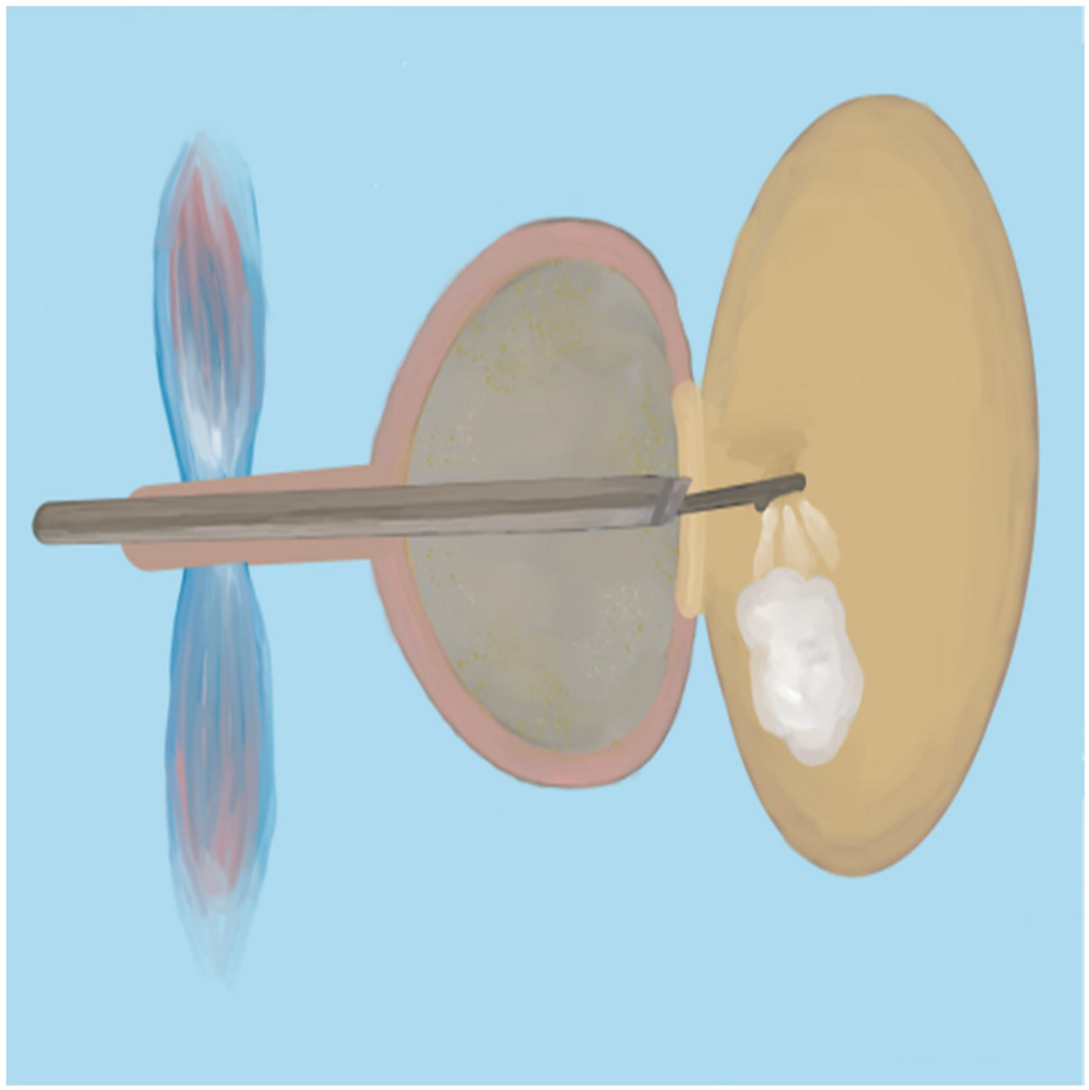
Morcellate the gland.

Since bipolar plasma devices, holmium lasers, and thulium lasers are all currently mainstream equipment for BPH surgical treatment, how to select the appropriate equipment based on various patient factors has become a issue requiring preoperative consideration. In terms of surgical efficiency, holmium lasers and thulium lasers demonstrate superior enucleation efficiency compared to bipolar plasma enucleation, particularly for large-volume prostates. Therefore, it is advisable to choose holmium lasers or thulium lasers for the surgical treatment of patients with large-volume BPH (65). Meanwhile, bipolar plasma devices utilize the same system as the plasma transurethral resection of the prostate (TURP) instruments that surgeons have commonly used in the past. This familiarity makes them easier to adopt, significantly shortening the learning curve. Furthermore, these devices enable surgeons to transition to the TURP surgical method at any time when confronted with complex situations (66). Regarding the learning curve for B-TUEP, although individual physicians vary in learning ability and proficiency, data indicate that clinicians performing B-TUEP for the first time can generally achieve proficiency after approximately 20 surgeries. This proficiency reduces the likelihood of converting to TURP during the operation, thereby ensuring optimal surgical outcomes and a low incidence of postoperative complications (26). In contrast, the learning curve for holmium lasers is longer, requiring more than 30 cases to achieve proficiency (67, 68). This results in fewer surgeons skilled in laser surgery, which may explain why many surgeons prefer B-TUEP over HoLEP when choosing between these two surgical approaches. Additionally, since the majority of BPH patients are relatively elderly and some require long-term anticoagulant therapy due to other medical conditions, surgery in these cases is inevitably associated with a higher risk of bleeding, making intraoperative hemostasis more challenging. However, laser instruments can safely perform prostate enucleation even when patients are on anticoagulants (69). Many patients with benign prostatic hyperplasia may also have concurrent bladder stones. During B-TUEP surgery, if bladder stones are detected either preoperatively or intraoperatively, lithotripsy cannot be performed simultaneously. This necessitates that surgeons switch to a different set of surgical instruments for lithotripsy, which inevitably increases the patient's costs and complicates the surgeon's operational procedures. In contrast, laser devices, whether holmium or thulium lasers, can effectively manage bladder stones and complete the subsequent prostate enucleation using the same set of equipment, thereby greatly simplifying the procedure (70). As laser devices of the same category, thulium lasers exhibit superior performance compared to holmium lasers in terms of urinary incontinence control rate and hemoglobin reduction rate (19, 71). Whether utilizing plasma devices, holmium lasers, or thulium lasers, the surgical procedures for prostate enucleation remain fundamentally consistent. This paper outlines the key points of the three-lobe method of prostate enucleation as follows: First, a mark should be made at the one-third position of the proximal bladder end of the verumontanum, followed by an incision extending to the prostatic surgical capsule (Figure 1). Next, an incision is made at the 5 o'clock to 7 o'clock position of the bladder neck, extending outward to the marked position on the verumontanum and deepening to the prostatic surgical capsule. After connecting the resection planes, the median lobe of the prostate is enucleated and pushed into the bladder (Figure 2). The edge of the gland is marked at the 12 o'clock position, and the lateral lobe is gradually enucleated by moving upward from the 5 o'clock or 7 o'clock position and downward from the 12 o'clock position until the bladder neck is breached. The other lateral lobe is enucleated using the same technique (Figure 3). Finally, a dedicated morcellator is employed for negative pressure aspiration of the gland, which is then morcellated and removed (Figure 4). After the completion of the procedure, an appropriate urinary catheter is inserted, and bladder irrigation is maintained for a specified duration (72). Although prostate enucleation offers numerous advantages over TURP, surgeons must exercise caution throughout the procedure to prevent intraoperative and postoperative complications. Surgeons must clearly identify the anatomical planes during the operation and manipulate with care to minimize the risk of damaging the bladder neck, which could lead to bladder neck perforation or disruption (73). In addition, intraoperatively identifying the surgical capsule and achieving precise hemostasis are effective methods for ensuring a clear surgical field and reducing the risk of TURS caused by irrigation fluid entering the bloodstream through bleeding sites. Although some patients may experience transient urinary incontinence regardless of whether TURP or prostate enucleation is performed, the majority of these patients can achieve relief or even full recovery during the postoperative rehabilitation period. Furthermore, since all these procedures are transurethral, some patients may develop urethral strictures due to injury from surgical instruments or may experience bladder neck contracture postoperatively, which may necessitate subsequent urethral dilation or urethrotomy for symptom relief. Finally, as the seminal vesicles extend through the prostate, both the blunt dissection involved in enucleation and the thermal injury associated with resection during the surgical process may impact the seminal vesicles, potentially leading to sexual dysfunction (74). Certainly, some scholars have indicated that retrograde ejaculation resulting from surgery, which can lead to impaired sexual function, may also be a significant factor (75). Therefore, the pursuit of more minimally invasive and less traumatic surgical techniques to achieve improved surgical outcomes, enhance therapeutic effects for patients, alleviate their symptoms, improve their quality of life, and reduce surgical complications is a common goal shared by all surgeons. To this end, surgical methods for BPH are continuously evolving and being refined. With advancements in science and technology, the feasibility of integrating artificial intelligence (AI) into surgical procedures remains a topic of ongoing discussion. Nevertheless, surgeons continue to strive for better therapeutic outcomes by experimenting with various advanced instruments and equipment.

## 5 Limitations of the current literature

Currently, studies conducted by numerous scholars indicate that there is no standardized brand or specific surgical power designated for plasma devices, holmium lasers, or thulium lasers. It remains unclear whether these variations influence surgical efficacy and the incidence of complications. Additionally, the types and frequencies of postoperative complications reported in the literature are not entirely consistent, and many researchers have not calculated the overall incidence of these complications. Consequently, comparing postoperative complications to evaluate the advantages and disadvantages of the aforementioned instruments presents an unresolved challenge. Furthermore, no studies have assessed whether the occurrence of postoperative urinary incontinence in patients is attributable to the surgery itself or to pre-existing conditions, such as neurogenic bladder, which may have caused dysuria. This gap prevents a comprehensive evaluation of the impact of these factors on surgical outcomes. Lastly, our analysis focused solely on the data comparison of prostate enucleation surgeries utilizing three types of surgical instruments, without examining variations in enucleation techniques, such as the three-lobe method and the whole-lobe method. Whether the intraoperative operational procedures have an impact on surgical efficacy remains to be discussed.

## 6 Conclusion

In summary, ThuLEP is superior to HoLEP and B-TUEP in terms of operational precision, while B-TUEP excels in equipment popularity and ease of learning. When patients experience complications such as bladder stones, both thulium and holmium lasers can simplify the surgeon's procedures. However, since thulium lasers have been used in clinical practice for a shorter duration compared to the other two, additional data will be necessary in the future to support and verify the safety and efficacy of thulium laser surgery.

## References

[1] Bostwick D. The pathology of benign prostatic hyperplasia. In: Kirby P McConnell J Fitzpatrick J, editors. The Textbook of Benign Prostatic Hyperplasia. London: Isis Medical Media (2002). p. 5.

[2] Frick J Aulitzky W. Physiology of the prostate. Infection. (1991) 19(Suppl 3):S115–8. 10.1007/BF01643679 2055645

[3] Chughtai B Forde JC Thomas DD Laor L Hossack T Woo HH Benign prostatic hyperplasia. Nat Rev Dis Primers. (2016) 2(1):16031. 10.1038/nrdp.2016.31 27147135

[4] Speakman M Kirby R Doyle S Ioannou C. Burden of male lower urinary tract symptoms (LUTS) suggestive of benign prostatic hyperplasia (BPH)- focus on the UK. BJU Int. (2015) 115:508–19. 10.1111/bju.12745 24656222

[5] Haile ES Sotimehin AE Gill BC. Medical management of benign prostatic hyperplasia. Cleve Clin J Med. (2024) 91(3):163–70. 10.3949/ccjm.91a.23027 38429006

[6] Fogaing C Alsulihem A Campeau L Corcos J. Is early surgical treatment for benign prostatic hyperplasia preferable to prolonged medical therapy: pros and cons. Medicina. (2021) 57(4):368. 10.3390/medicina57040368 33918818 PMC8069902

[7] Rassweiler J Teber D Kuntz R Hofmann R. Complications of transurethral resection of the prostate (TURP)–incidence, management, and prevention. Eur Urol. (2006) 50(5):969–79; discussion 980. 10.1016/j.eururo.2005.12.042 16469429

[8] Miernik A Gratzke C. Current treatment for benign prostatic hyperplasia. Dtsch Arztebl Int. (2020) 117(49):843–54. 10.3238/arztebl.2020.0843 33593479 PMC8021971

[9] Erturhan S Erbagci A Seckiner I Yagci F Ustun A. Plasmakinetic resection of the prostate versus standard transurethral resection of the prostate: a prospective randomized trial with 1-year follow-up. Prostate Cancer Prostatic Dis. (2007) 10(1):97–100. 10.1038/sj.pcan.4500907 16926854

[10] Zeng XT Jin YH Liu TZ Chen FM Ding DG Fu M Clinical practice guideline for transurethral plasmakinetic resection of prostate for benign prostatic hyperplasia (2021 edition). Mil Med Res. (2022) 9(1):14. 10.1186/s40779-022-00371-6 35361280 PMC8974007

[11] Zhang Y Yuan P Ma D Gao X Wei C Liu Z Efficacy and safety of enucleation vs. resection of prostate for treatment of benign prostatic hyperplasia: a meta-analysis of randomized controlled trials. Prostate Cancer Prostatic Dis. (2019) 22(4):493–508. 10.1038/s41391-019-0135-4 30816336

[12] Zarrabi A Gross AJ. The evolution of lasers in urology. Ther Adv Urol. (2011) 3(2):81–9. 10.1177/1756287211400494 21869908 PMC3150070

[13] Kuntz RM Lehrich K Ahyai SA. Holmium laser enucleation ofthe prostate versus open prostatectomy for prostates greater than 100 grams: 5-year follow-up results of a randomised clinical trial. Eur Urol. (2008) 53(1):160–6. 10.1016/j.eururo.2007.08.036 17869409

[14] Kuebker JM Miller NL. Holmium laser enucleation of the prostate: patient selection and outcomes. Curr Urol Rep. (2017) 18(12):96. 10.1007/s11934-017-0746-z 29046983

[15] de Figueiredo FCA Cracco CM de Marins RL Scoffone CM. Holmium laser enucleation of the prostate: problem-based evolution of the technique. Andrologia. (2020) 52(8):e13582. 10.1111/and.13582 32267013

[16] Bach T Muschter R Sroka R Gravas S Skolarikos A Herrmann TR Laser treatment of benign prostatic obstruction: basics and physical differences. Eur Urol. (2012) 61(2):317–25. 10.1016/j.eururo.2011.10.009 22033173

[17] Enikeev D Taratkin M. Thulium fiber laser: bringing lasers to a whole new level. Eur Urol Open Sci. (2022) 48:31–3. 10.1016/j.euros.2022.07.007 36588770 PMC9795521

[18] Jackson SD Lauto A. Diode-pumped fiber lasers: a new clinical tool? Lasers Surg Med. (2002) 30(3):184–90. 10.1002/lsm.10023 11891737

[19] Hartung FO Kowalewski KF von Hardenberg J Worst TS Kriegmair MC Nuhn P Holmium versus thulium laser enucleation of the prostate: a systematic review and meta-analysis of randomized controlled trials. Eur Urol Focus Mar. (2022) 8(2):545–54. 10.1016/j.euf.2021.03.024 33840611

[20] Taratkin M Azilgareeva C Cacciamani GE Enikeev D. Thulium fiber laser in urology: physics made simple. Curr Opin Urol. (2022) 32(2):166–72. 10.1097/MOU.0000000000000967 34954703

[21] Hiraoka Y Akimoto M. Transurethral enucleation of benign prostatic hyperplasia. J Urol. (1989) 142(5):1247–50. 10.1016/S0022-5347(17)39047-X 2478727

[22] Neill MG Gilling PJ Kennett KM Frampton CM Westenberg AM Fraundorfer MR Randomized trial comparing holmium laser enucleation of prostate with plasmakinetic enucleation of prostate for treatment of benign prostatic hyperplasia. Urology. (2006) 68(5):1020–4. 10.1016/j.urology.2006.06.021 17095078

[23] Reddy SK Utley V Gilling PJ. The evolution of endoscopic prostate enucleation: a historical perspective. Andrologia. (2020) 52(8):e13673. 10.1111/and.13673 32557842

[24] Rao JM Yang JR Ren YX He J Ding P Yang JH. Plasmakinetic enucleation of the prostate versus transvesical open prostatectomy for benign prostatic hyperplasia >80ml: 12-month follow-up results of a randomized clinical trial. Urology. (2013) 82:176–81. 10.1016/j.urology.2013.02.032 23601443

[25] Arcaniolo D Manfredi C Veccia A Herrmann TRW Lima E Mirone V Bipolar endoscopic enucleation versus bipolar transurethral resection of the prostate: an ESUT systematic review and cumulative analysis. World J Urol. (2020) 38(5):1177–86. 10.1007/s00345-019-02890-9 31346761

[26] Ramesmayer C Deininger S Pyrgidis N Lusuardi L Kunit T Pallauf M The early learning curve of the bipolar enucleation of the prostate: a multicenter cohort study. World J Urol. (2024) 42(1):478. 10.1007/s00345-024-05183-y 39115714 PMC11310227

[27] Huang SW Tsai CY Tseng CS Shih MC Yeh YC Chien KL Comparative efficacy and safety of new surgical treatments for benign prostatic hyperplasia: systematic review and network meta-analysis. Br Med J. (2019) 367:l5919. 10.1136/bmj.l5919 31727627 PMC7223639

[28] Lin Y Wu X Xu A Ren R Zhou X Wen Y Transurethral enucleation of the prostate versus transvesical open prostatectomy for large benign prostatic hyperplasia: a systematic review and meta-analysis of randomized controlled trials. World J Urol. (2016) 34(9):1207–19. 10.1007/s00345-015-1735-9 26699627

[29] Zhang F Shao Q Herrmann TR Tian Y Zhang Y. Thulium laser versus holmium laser transurethral enucleation of the prostate: 18-month follow-up data of a single center. Urology. (2012) 79(4):869–74. 10.1016/j.urology.2011.12.018 22342411

[30] Sun F Yao H Bao X Wang X Wang D Zhang D The efficacy and safety of HoLEP for benign prostatic hyperplasia with large volume: a systematic review and meta-analysis. Am J Mens Health. (2022) 16(4):15579883221113203. 10.1177/15579883221113203 35864746 PMC9310232

[31] Li J Cao D Huang Y Meng C Peng L Xia Z Holmium laser enucleation versus bipolar transurethral enucleation for treating benign prostatic hyperplasia, which one is better? Aging Male. (2021) 24(1):160–70. 10.1080/13685538.2021.2014807 34895034

[32] Chen YY Hua WX Huang YH Shen XY You JN Ding X. The safety and efficacy of five surgical treatments in prostate enucleation: a network meta-analysis. BMC Urol. (2024) 24(1):128. 10.1186/s12894-024-01517-5 38886739 PMC11181543

[33] Scoffone CM Cracco CM. High-power HoLEP: no thanks!. World J Urol. (2018) 36(5):837–8. 10.1007/s00345-018-2186-x 29374842

[34] Das AK Han TM Hardacker TJ. Holmium laser enucleation of the prostate (HoLEP): size-independent gold standard for surgical management of benign prostatic hyperplasia. Can J Urol. (2020) 27:44–50. 32876002

[35] Habib E Abdallah MF ElSheemy MS Badawy MH Nour HH Kamal AM Holmium laser enucleation versus bipolar resection in the management of large-volume benign prostatic hyperplasia: a randomized controlled trial. Int J Urol. (2022) 29(2):128–35. 10.1111/iju.14737 34788900

[36] Chen F Chen Y Zou Y Wang Y Wu X Chen M. Comparison of holmium laser enucleation and transurethral resection of prostate in benign prostatic hyperplasia: a systematic review and meta-analysis. J Int Med Res. (2023) 51(8):3000605231190763. 10.1177/03000605231190763 37561537 PMC10416666

[37] Deuker M Rührup J Karakiewicz PI Welte M Kluth LA Banek S Holmium laser enucleation of the prostate: efficacy, safety and preoperative management in patients presenting with anticoagulation therapy. World J Urol. (2021) 39(4):1219–26. 10.1007/s00345-020-03272-2 32488362 PMC8124040

[38] Fallara G Capogrosso P Schifano N Costa A Candela L Cazzaniga W Ten-year follow-up results after holmium laser enucleation of the prostate. Eur Urol Focus. (2021) 7(3):612–7. 10.1016/j.euf.2020.05.012 32576532

[39] Ditonno F Bianchi A Fumanelli F Brancelli C Malandra S Rizzetto R The learning curve for holmium laser enucleation of the prostate: a single-center analysis of surgical and functional outcomes. J Endourol. (2024) 38(11):1226–33. 10.1089/end.2024.0422 39135470

[40] Kosiba M Hoeh B Welte MN Krimphove MJ Vitucci K Lindemann N Learning curve and functional outcomes after laser enucleation of the prostate for benign prostate hyperplasia according to surgeon’s caseload. World J Urol. (2022) 40(12):3007–13. 10.1007/s00345-022-04177-y 36289106 PMC9712403

[41] Schiavina R Bianchi L Giampaoli M Borghesi M Dababneh H Chessa F Holmium laser prostatectomy in a tertiary Italian center: a prospective cost analysis in comparison with bipolar TURP and open prostatectomy. Arch Ital Urol Androl. (2020) 92(2). 10.4081/aiua.2020.2.82 32597105

[42] Franz J Suarez-Ibarrola R Pütz P Sigle A Lusuardi L Netsch C Morcellation after endoscopic enucleation of the prostate: efficiency and safety of currently available devices. Eur Urol Focus. (2022) 8(2):532–44. 10.1016/j.euf.2021.03.021 33858810

[43] Zebić N Terzić V Krajina V. Thulium:YAG laser enucleation of the prostate (ThuLEP) - our experience in 246 patients. Acta Clin Croat. (2023) 62(Suppl2):104–9. 10.20471/acc.2023.62.s2.14 38966028 PMC11221234

[44] Meng C Peng L Li J Li J Li Y Yang J Comparison of enucleation between thulium laser and holmium laser for benign prostatic hyperplasia: a systematic review and meta-analysis. Asian J Surg. (2022) 45(2):689–97. 10.1016/j.asjsur.2021.07.045 34384678

[45] Enikeev D Glybochko P Rapoport L Gahan J Gazimiev M Spivak L A randomized trial comparing the learning curve of 3 endoscopic enucleation techniques (HoLEP, ThuFLEP, and MEP) for BPH using mentoring approach-initial results. Urology. (2018) 121:51–7. 10.1016/j.urology.2018.06.045 30053397

[46] Bozzini G Berti L Maltagliati M Besana U Calori A Müller A Ejaculation-sparing thulium laser enucleation of the prostate (ES-ThuLEP): outcomes on a large cohort. World J Urol. (2021) 39(6):2029–35. 10.1007/s00345-020-03442-2 32929626

[47] Saredi G Pirola GM Pacchetti A Lovisolo JA Borroni G Sembenini F Evaluation of the learning curve for thulium laser enucleation of the prostate with the aid of a simulator tool but without tutoring: comparison of two surgeons with different levels of endoscopic experience. BMC Urol. (2015) 15(1):1–7. 10.1186/s12894-015-0045-2 PMC4459696 26055885

[48] Xiao KW Zhou L He Q Gao XS Chen G Ma YC Enucleation of the prostate for benign prostatic hyperplasia thulium laser versus holmium laser: a systematic review and meta-analysis. Lasers Med Sci. (2019) 34(4):815–26. 10.1007/s10103-018-02697-x 30604345

[49] Moore J Chavez A Narang G Bogle J Stern K. Operating room noise hazards during laser lithotripsy: a comparison between the thulium fiber and holmium laser platforms. World J Urol. (2022) 40(3):801–5. 10.1007/s00345-021-03897-x 35059787

[50] Spinos T Tatanis V Peteinaris A Somani B Kartalas Goumas I Liatsikos E Thulium fiber laser enucleation of the prostate: a systematic review of the current outcomes. Minerva Urol Nephrol. (2024) 76(2):157–65. 10.23736/S2724-6051.24.05654-4 38742551

[51] Uroweb. EAU guidelines: management of non-neurogenic Male LUTS. (n.d.) [Internet]. Available online at: https://uroweb.org/guideline/treatment-ofnon-neurogenic-male-luts/#5 (Accessed February 5, 2021).

[52] American Urological Association. Benign prostatic hyperplasia (bph) guideline. (n.d.) [Internet]. Available online at: https://www.auanet.org/guidelines/guidelines/benign-prostatic-hyperplasia-(bph)-guideline (Accessed July 27, 2021).

[53] Lerner LB McVary KT Barry MJ Bixler BR Dahm P Das AK Management of lower urinary tract symptoms attributed to benign prostatic hyperplasia: AUA GUIDELINE PART II—surgical evaluation and treatment. J Urol. (2021) 206(4):818–26. 10.1097/JU.0000000000002184 34384236

[54] Priest R Garzotto M Kaufman J. Benign prostatic hyperplasia: a brief overview of pathogenesis, diagnosis, and therapy. Tech Vasc Interv Radiol. (2012) 15(4):261–4. 10.1053/j.tvir.2012.10.001 23244721

[55] Kim M Jeong CW Oh SJ. Effect of preoperative urodynamic detrusor underactivity on transurethral surgery for benign prostatic hyperplasia: a systematic review and meta-analysis. J Urol. (2018) 199(1):237–44. 10.1016/j.juro.2017.07.079 28760632

[56] Lerner LB McVary KT Barry MJ Bixler BR Dahm P Das AK Management of lower urinary tract symptoms attributed to benign prostatic hyperplasia: AUA GUIDELINE PART I-initial work-up and medical management. J Urol. (2021) 206(4):806–17. 10.1097/JU.0000000000002183; Erratum in: J Urol. 2021 Nov;206(5):1339. doi: 10.1097/JU.0000000000002231. 34384237

[57] Mu X Guo L Guo Z Zhang L Wang S. Diode laser enucleation vs. bipolar transurethral enucleation of prostate for benign prostatic hyperplasia: a retrospective comparative study with three-year follow up. Arch Esp Urol. (2023) 76(2):161–8. 10.56434/j.arch.esp.urol.20237602.18 37139622

[58] Lusuardi L Mitterberger M Hruby S Kunit T Kloss B Engelhardt PF Update on the use of diode laser in the management of benign prostate obstruction in 2014. World J Urol. (2015) 33(4):555–62. 10.1007/s00345-014-1327-0 24859776

[59] Yang SS Hsieh CH Lee YS Chang SJ. Diode laser (980nm) enucleation of the prostate: a promising alternative to transurethral resection of the prostate. Lasers Med Sci. (2013) 28(2):353–60. 10.1007/s10103-011-1046-3 22282073

[60] Elshal AM Elmansy HM Elhilali MM. Two laser ablation techniques for a prostate less than 60ml: lessons learned 70 months after a randomized controlled trial. Urology. (2013) 82:416–22. 10.1016/j.urology.2013.02.074 23791215

[61] Rieken M Bachmann A. Laser treatment of benign prostate enlargement–which laser for which prostate? Nat Rev Urol. (2014) 11(3):142–52. 10.1038/nrurol.2014.23 24595121

[62] Li KP Chen SY Yang L. Laparoscopic simple prostatectomy versus robot-assisted simple prostatectomy for large benign prostatic hyperplasia: a systematic review and meta-analysis of comparative trials. J Robot Surg. (2023) 17(2):351–64. 10.1007/s11701-022-01460-3 36272059

[63] Lombardo R Zarraonandia Andraca A Plaza Alonso C González-Dacal JA Rodríguez Núñez H Barreiro Mallo A Laparoscopic simple prostatectomy vs bipolar plasma enucleation of the prostate in large benign prostatic hyperplasia: a two-center 3-year comparison. World J Urol. (2021) 39(7):2613–9. 10.1007/s00345-020-03512-5 33175211 PMC8332603

[64] Pyrgidis N Mykoniatis I Lusuardi L Schulz GB Sokolakis I Stief C Enucleation of the prostate as retreatment for recurrent or residual benign prostatic obstruction: a systematic review and a meta-analysis. Prostate Cancer Prostatic Dis. (2023) 26(4):693–701. 10.1038/s41391-023-00677-z 37193777

[65] Sandhu JS Bixler BR Dahm P Goueli R Kirkby E Stoffel JT Management of lower urinary tract symptoms attributed to benign prostatic hyperplasia (BPH): AUA guideline amendment 2023. J Urol. (2024) 211(1):11–9. 10.1097/JU.0000000000003698 37706750

[66] Patard PM Roumiguie M Sanson S Beauval JB Huyghe E Soulié M Endoscopic enucleation for prostate larger than 60ml: comparison between holmium laser enucleation and plasmakinetic enucleation. World J Urol. (2021) 39(6):2011–8. 10.1007/s00345-020-03382-x 32719929

[67] Robert G Cornu J-N Fourmarier M Saussine C Descazeaud A Azzouzi A-R Multicentre prospective evaluation of the learning curve of holmium laser enucleation of the prostate (HoLEP). BJU Int. (2016) 117:495–9. 10.1111/bju.13124 25781490

[68] Brunckhorst O Ahmed K Nehikhare O Marra G Challacombe B Popert R. Evaluation of the learning curve for holmium laser enucleation of the prostate using multiple outcome measures. Urology. (2015) 86:824–9. 10.1016/j.urology.2015.07.021 26254171

[69] Rivera M Krambeck A Lingeman J. Holmium laser enucleation of the prostate in patients requiring anticoagulation. Curr Urol Rep. (2017) 18(10):77. 10.1007/s11934-017-0727-2 28780634

[70] Li Y Yang Y Chen J Li Z Song G Chen J Thulium laser enucleation of the prostate plus thulium fiber laser therapy for benign prostatic hyperplasia combined with bladder stones. Wideochir Inne Tech Maloinwazyjne. (2024) 19(3):370–6. 10.20452/wiitm.2024.17897 40125253 PMC11927542

[71] Bozzini G Berti L Aydoğan TB Maltagliati M Roche JB Bove P A prospective multicenter randomized comparison between holmium laser enucleation of the prostate (HoLEP) and thulium laser enucleation of the prostate (ThuLEP). World J Urol. (2021) 39(7):2375–82. 10.1007/s00345-020-03468-6 32997262

[72] Herrmann TR Bach T Imkamp F Georgiou A Burchardt M Oelke M Thulium laser enucleation of the prostate (ThuLEP): transurethral anatomical prostatectomy with laser support. Introduction of a novel technique for the treatment of benign prostatic obstruction. World J Urol. (2010) 28(1):45–51. 10.1007/s00345-009-0503-0 20063164

[73] da Silva RD Bidikov L Michaels W Gustafson D Molina WR Kim FJ. Bipolar energy in the treatment of benign prostatic hyperplasia: a current systematic review of the literature. Can J Urol. (2015) 22(Suppl. 1):30–44. 26497342

[74] Kim SH Yang HK Lee HE Paick JS Oh SJ. HoLEP does not affect the overall sexual function of BPH patients: a prospective study. Asian J Androl. (2014) 16(6):873–7. 10.4103/1008-682X.132469 25038179 PMC4236332

[75] Xu YW Liu CX Zheng SB Li HL Fang P Chen BS. Transurethral enucleation of the prostate for treatment of benign prostatic hyperplasia in patients less than 50 years old. Nan Fang Yi Ke Da Xue Xue Bao. (2010) 30:2708–10. 21177187

